# (*E*)-3-(4-Eth­oxy­phen­yl)-1-(2-hy­droxy­phen­yl)prop-2-en-1-one

**DOI:** 10.1107/S1600536810032514

**Published:** 2010-08-18

**Authors:** Jirapa Horkaew, Suchada Chantrapromma, Nisakorn Saewan, Hoong-Kun Fun

**Affiliations:** a Crystal Materials Research Unit, Department of Chemistry, Faculty of Science, Prince of Songkla University, Hat-Yai, Songkhla 90112, Thailand; b School of Cosmetic Science, Mae Fah Luang University, Muang, Chiang Rai 57100, Thailand; c X-ray Crystallography Unit, School of Physics, Universiti Sains Malaysia, 11800 USM, Penang, Malaysia

## Abstract

In the title compound, C_17_H_16_O_3_, the carbonyl group is in an *s-cis* configuration with respect to the olefinic double bond. The dihedral angle between the two benzene rings is 2.85 (3)°. The prop-2-en-1-one bridge makes dihedral angles of 4.77 (4) and 4.15 (4)°, respectively, with the 2-hy­droxy­phenyl and 4-eth­oxy­phenyl rings. The eth­oxy group is coplanar with the attached phenyl ring [C_ar_—O—C—C = 179.72 (5)°]. An intra­molecular O—H⋯O hydrogen bond generates an *S*(6) ring motif. In the crystal structure, mol­ecules are stacked in an anti­parallel manner to form columns along the *b* axis. The columnar structure is stabilized by C—H⋯π inter­actions involving the 2-hy­droxy­phenyl ring.

## Related literature

For background on the applications of chalcones, see: Jun *et al.* (2007 ▶); Nowakowska (2007 ▶); Patil & Dharmaprakash (2008 ▶); Saydam *et al.* (2003 ▶); Svetlichny *et al.* (2007 ▶); Tewtrakul *et al.* (2003 ▶). For bond-length data, see: Allen *et al.* (1987 ▶). For hydrogen-bond motifs, see: Bernstein *et al.* (1995 ▶). For related structures, see: Fun *et al.* (2008 ▶); Patil *et al.* (2007 ▶). For the stability of the temperature controller used in the data collection, see: Cosier & Glazer (1986 ▶).
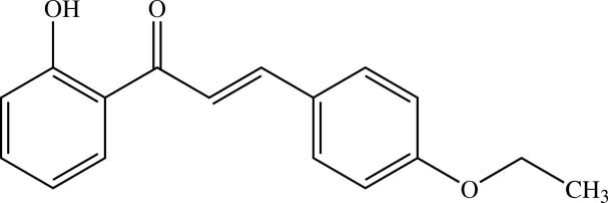


## Experimental

### 

#### Crystal data

C_17_H_16_O_3_
*M* *_r_* = 268.30
Triclinic, 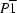

*a* = 6.8305 (2) Å
*b* = 6.8790 (2) Å
*c* = 14.8188 (3) Å
α = 88.533 (1)°
β = 80.380 (1)°
γ = 77.469 (1)°
*V* = 670.11 (3) Å^3^
*Z* = 2
Mo *K*α radiation
μ = 0.09 mm^−1^
*T* = 100 K
0.60 × 0.38 × 0.36 mm

#### Data collection

Bruker APEXII CCD area-detector diffractometer
Absorption correction: multi-scan (*SADABS*; Bruker, 2005 ▶) *T*_min_ = 0.948, *T*_max_ = 0.969
24772 measured reflections
5849 independent reflections
5179 reflections with *I* > 2σ(*I*)
*R*_int_ = 0.020

#### Refinement

*R*[*F* ^2^ > 2σ(*F* ^2^)] = 0.041
*wR*(*F* ^2^) = 0.124
*S* = 1.04
5848 reflections
186 parameters
H atoms treated by a mixture of independent and constrained refinement
Δρ_max_ = 0.39 e Å^−3^
Δρ_min_ = −0.44 e Å^−3^

### 

Data collection: *APEX2* (Bruker, 2005 ▶); cell refinement: *SAINT* (Bruker, 2005 ▶); data reduction: *SAINT*; program(s) used to solve structure: *SHELXTL* (Sheldrick, 2008 ▶); program(s) used to refine structure: *SHELXTL*; molecular graphics: *SHELXTL*; software used to prepare material for publication: *SHELXTL* and *PLATON* (Spek, 2009 ▶).

## Supplementary Material

Crystal structure: contains datablocks global, I. DOI: 10.1107/S1600536810032514/ci5143sup1.cif

Structure factors: contains datablocks I. DOI: 10.1107/S1600536810032514/ci5143Isup2.hkl

Additional supplementary materials:  crystallographic information; 3D view; checkCIF report

## Figures and Tables

**Table 1:** Hydrogen-bond geometry (Å, °) *Cg*1 is the centroid of the C1–C6 ring.

*D*—H⋯*A*	*D*—H	H⋯*A*	*D*⋯*A*	*D*—H⋯*A*
O2—H1*O*2⋯O1	0.93 (2)	1.66 (2)	2.5113 (7)	151 (1)
C16—H16*A*⋯*Cg*1^i^	0.97	2.70	3.5762 (7)	151
C16—H16*B*⋯*Cg*1^ii^	0.97	2.66	3.5339 (7)	151

Symmetry codes: (i) 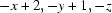; (ii) 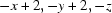.

**Fig. 1.**
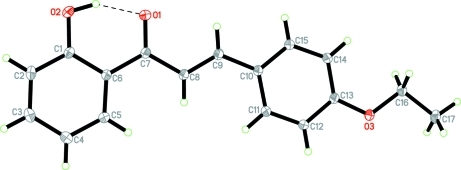
The molecular structure of the title compound, showing 50% probability displacement ellipsoids and the atom-numbering scheme. Intramolecular O—H···O hydrogen bond is shown as a dashed line.

**Fig. 2.**
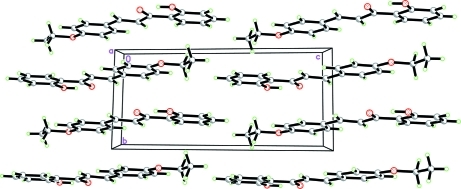
The crystal packing of the title compound viewed along the a axis, showing antiparallel stacked columns running along the b axis.

## supplementary crystallographic information

### Comment

Chalcones is an interesting class of compounds which have been reported to posses various useful properties such as non-linear optical (NLO) (Patil & Dharmaprakash, 2008) and fluorescent properties (Svetlichny *et al.*, 2007). Synthetic and naturally occurring chalcones have been found to exhibit many useful biological activities, including anti-inflammatory, antileishmanial, antimicrobial, antioxidant (Nowakowska, 2007; Saydam *et al.*, 2003), HIV-1 protease inhibitory (Tewtrakul *et al.*, 2003) and tyrosinase inhibitory (Jun *et al.*, 2007) activities. Our on going research on NLO properties and bioactivities of the synthetic chalcones led us to synthesize the title chalcone in order to study its NLO properties, antibacterial and tyrosinase inhibitory activities. The results show that the title compound, (I), crystallized in centrosymmetric *P*1 space group which prohibits the second order NLO properties. Our biological testing found that (I) was inactive against the tested bacteria which are Gram-positive bacteria *i.e. Bacillus subtilis*, *Enterococcus faecalis*, *Staphylococcus aureus*, Methicillin-Resistant *Staphylococcus aureus* and Vancomycin-Resistant *Enterococcus faecalis* and Gram-negative bacteria *i.e. Pseudomonas aeruginosa*, *Salmonella typhi* and *Shigella sonnei*. Nevertheless (I) shows moderate tyrosinase inhibitory activity which will be reported elsewhere with some other chalcones. Herein the crystal structure of (I) is reported.

The title molecule (Fig. 1) exists in an *E* configuration with respect to the C8═C9 ethenyl bond [1.3475 (8) Å]; the C7–C8–C9–C10 torsion angle is -178.73 (6)°. The molecule is almost planar with the dihedral angle between the 2-hydroxyphenyl and 4-ethoxyphenyl rings being 2.85 (3)°. The substituted ethoxy group is coplanar with the attached phenyl ring with the torsion angle C13–O3–C16–C17 = 179.72 (5)°. The prop-2-en-1-one unit (C7–C9/O1) is planar [*r.m.s.* deviation 0.0098 (1) Å] and the torsion angle O1–C7–C8–C9 being 3.15 (10)°. This bridge makes dihedral angles of 4.77 (4) and 4.15 (4)° with the 2-hydroxyphenyl and 4-ethoxyphenyl rings, respectively. Intramolecular O2—H1O2···O1 hydrogen bond generates an S(6) ring motif (Fig. 1) (Bernstein *et al.*, 1995) which helps to stabilize the planarity of the chalcone skeleton. The bond distances are of normal values (Allen *et al.*, 1987) and are comparable with those observed in related structures (Fun *et al.*, 2008; Patil *et al.*, 2007).

In the crystal packing, the molecules are stacked in an antiparallel manner into columns along the *b* axis (Fig. 2). This arrangement is stabilized by C—H···π interactions (Table 1) involving the hydroxy phenyl ring. In addition C···O short contacts [3.2894 (8)–3.4003 (9) Å] were also observed.

### Experimental

The title compound was synthesized by the condensation of 4-ethoxycarboxaldehyde (0.30 g, 2 mmol) with 2-hydroxyacetophenone (0.24 ml, 2 mmol) in ethanol (40 ml) in the presence of 30% aqueous NaOH (5 ml) at room temperature. After stirring for 3 h, 20% H_2_SO_4_(aq) (5 ml) was added dropwise into the solution. After the reaction mixture was kept at room temperature for a day, a yellow solid appeared and was then collected by filtration, washed with acetone and dried in air. Yellow block-shaped single crystals of the title compound suitable for X-ray structure determination were recrystalized from acetone by slow evaporation of the solvent at room temperature after several days (m.p. 387–388 K).

### Refinement

Hydroxyl H atom was located in a difference map and refined isotropically. The remaining H atoms were placed in calculated positions, with C–H = 0.93 Å, *U*_iso_ = 1.2*U*_eq_(C) for aromatic and CH, C–H = 0.96 Å, *U*_iso_ = 1.2*U*_eq_(C) for CH_2_, and C–H = 0.97 Å, *U*_iso_ = 1.5*U*_eq_(C) for CH_3_ atoms. A rotating group model was used for the methyl groups.

### Crystal data

**Table:**

C_17_H_16_O_3_	*Z* = 2
*M**_r_* = 268.30	*F*(000) = 284
Triclinic, *P*1	*D*_x_ = 1.330 Mg m^−^^3^
Hall symbol: -P 1	Melting point = 387–388 K
*a* = 6.8305 (2) Å	Mo *K*α radiation, λ = 0.71073 Å
*b* = 6.8790 (2) Å	Cell parameters from 5849 reflections
*c* = 14.8188 (3) Å	θ = 1.4–35.0°
α = 88.533 (1)°	µ = 0.09 mm^−^^1^
β = 80.380 (1)°	*T* = 100 K
γ = 77.469 (1)°	Block, yellow
*V* = 670.11 (3) Å^3^	0.60 × 0.38 × 0.36 mm

### Data collection

**Table:**

Bruker APEXII CCD area-detector diffractometer	5849 independent reflections
Radiation source: sealed tube	5179 reflections with *I* > 2σ(*I*)
graphite	*R*_int_ = 0.020
φ and ω scans	θ_max_ = 35.0°, θ_min_ = 1.4°
Absorption correction: multi-scan (*SADABS*; Bruker, 2005)	*h* = −11→11
*T*_min_ = 0.948, *T*_max_ = 0.969	*k* = −10→11
24772 measured reflections	*l* = −23→23

### Refinement

**Table:**

Refinement on *F*^2^	Primary atom site location: structure-invariant direct methods
Least-squares matrix: full	Secondary atom site location: difference Fourier map
*R*[*F*^2^ > 2σ(*F*^2^)] = 0.041	Hydrogen site location: inferred from neighbouring sites
*wR*(*F*^2^) = 0.124	H atoms treated by a mixture of independent and constrained refinement
*S* = 1.04	*w* = 1/[σ^2^(*F*_o_^2^) + (0.0744*P*)^2^ + 0.1118*P*] where *P* = (*F*_o_^2^ + 2*F*_c_^2^)/3
5848 reflections	(Δ/σ)_max_ = 0.001
186 parameters	Δρ_max_ = 0.39 e Å^−^^3^
0 restraints	Δρ_min_ = −0.44 e Å^−^^3^

### Special details

**Table:**

Experimental. The crystal was placed in the cold stream of an Oxford Cryosystems Cobra open-flow nitrogen cryostat (Cosier & Glazer, 1986) operating at 100.0 (1) K.
Geometry. All esds (except the esd in the dihedral angle between two l.s. planes) are estimated using the full covariance matrix. The cell esds are taken into account individually in the estimation of esds in distances, angles and torsion angles; correlations between esds in cell parameters are only used when they are defined by crystal symmetry. An approximate (isotropic) treatment of cell esds is used for estimating esds involving l.s. planes.
Refinement. Refinement of *F*^2^ against ALL reflections. The weighted *R*-factor *wR* and goodness of fit *S* are based on *F*^2^, conventional *R*-factors *R* are based on *F*, with *F* set to zero for negative *F*^2^. The threshold expression of *F*^2^ > σ(*F*^2^) is used only for calculating *R*-factors(gt) *etc*. and is not relevant to the choice of reflections for refinement. *R*-factors based on *F*^2^ are statistically about twice as large as those based on *F*, and *R*- factors based on ALL data will be even larger.

### Fractional atomic coordinates and isotropic or equivalent isotropic displacement parameters (Å^2^)

**Table:**

	*x*	*y*	*z*	*U*_iso_*/*U*_eq_	
O1	1.46060 (7)	0.63044 (8)	0.13054 (3)	0.01858 (10)	
O2	1.59166 (7)	0.61854 (8)	0.27959 (4)	0.01890 (11)	
H1O2	1.589 (2)	0.614 (2)	0.2173 (11)	0.052 (4)*	
O3	0.61756 (7)	0.84310 (8)	−0.23045 (3)	0.01749 (10)	
C1	1.39580 (9)	0.65747 (9)	0.32040 (4)	0.01353 (11)	
C2	1.35615 (10)	0.66707 (10)	0.41620 (4)	0.01724 (12)	
H2A	1.4636	0.6474	0.4490	0.021*	
C3	1.15843 (11)	0.70553 (11)	0.46221 (5)	0.02141 (13)	
H3A	1.1333	0.7123	0.5258	0.026*	
C4	0.99566 (11)	0.73437 (12)	0.41364 (5)	0.02221 (14)	
H4A	0.8626	0.7594	0.4448	0.027*	
C5	1.03359 (10)	0.72550 (10)	0.31877 (4)	0.01683 (12)	
H5A	0.9248	0.7447	0.2869	0.020*	
C6	1.23305 (9)	0.68816 (9)	0.26988 (4)	0.01237 (10)	
C7	1.27920 (9)	0.67709 (9)	0.16865 (4)	0.01267 (10)	
C8	1.11547 (9)	0.72198 (9)	0.11403 (4)	0.01350 (11)	
H8A	0.9811	0.7630	0.1425	0.016*	
C9	1.16056 (9)	0.70358 (9)	0.02217 (4)	0.01346 (11)	
H9A	1.2972	0.6595	−0.0024	0.016*	
C10	1.01927 (9)	0.74506 (9)	−0.04275 (4)	0.01229 (10)	
C11	0.80665 (9)	0.79158 (9)	−0.01582 (4)	0.01357 (11)	
H11A	0.7517	0.7999	0.0461	0.016*	
C12	0.67867 (9)	0.82501 (9)	−0.08002 (4)	0.01398 (11)	
H12A	0.5386	0.8566	−0.0611	0.017*	
C13	0.75873 (9)	0.81164 (9)	−0.17369 (4)	0.01315 (10)	
C14	0.96900 (9)	0.76806 (10)	−0.20234 (4)	0.01477 (11)	
H14A	1.0233	0.7606	−0.2643	0.018*	
C15	1.09656 (9)	0.73581 (9)	−0.13654 (4)	0.01429 (11)	
H15A	1.2367	0.7074	−0.1555	0.017*	
C16	0.68663 (9)	0.83005 (9)	−0.32736 (4)	0.01484 (11)	
H16A	0.7685	0.6985	−0.3445	0.018*	
H16B	0.7680	0.9278	−0.3465	0.018*	
C17	0.49767 (11)	0.87050 (11)	−0.37121 (5)	0.01895 (12)	
H17A	0.5358	0.8690	−0.4366	0.028*	
H17B	0.4155	0.9986	−0.3514	0.028*	
H17C	0.4217	0.7696	−0.3536	0.028*	

### Atomic displacement parameters (Å^2^)

**Table:**

	*U*^11^	*U*^22^	*U*^33^	*U*^12^	*U*^13^	*U*^23^
O1	0.01206 (19)	0.0280 (3)	0.0151 (2)	−0.00354 (17)	−0.00144 (15)	−0.00028 (17)
O2	0.01199 (19)	0.0265 (2)	0.0186 (2)	−0.00291 (17)	−0.00501 (16)	−0.00171 (18)
O3	0.01373 (19)	0.0271 (3)	0.01164 (19)	−0.00273 (17)	−0.00437 (14)	0.00013 (16)
C1	0.0135 (2)	0.0129 (2)	0.0151 (2)	−0.00301 (18)	−0.00482 (18)	0.00005 (18)
C2	0.0209 (3)	0.0179 (3)	0.0145 (2)	−0.0041 (2)	−0.0076 (2)	0.00091 (19)
C3	0.0244 (3)	0.0268 (3)	0.0130 (2)	−0.0054 (2)	−0.0034 (2)	0.0009 (2)
C4	0.0174 (3)	0.0329 (4)	0.0149 (3)	−0.0044 (2)	−0.0001 (2)	0.0007 (2)
C5	0.0130 (2)	0.0228 (3)	0.0145 (2)	−0.0035 (2)	−0.00232 (19)	0.0006 (2)
C6	0.0120 (2)	0.0133 (2)	0.0122 (2)	−0.00281 (17)	−0.00315 (17)	0.00025 (17)
C7	0.0127 (2)	0.0131 (2)	0.0129 (2)	−0.00335 (17)	−0.00318 (17)	0.00039 (17)
C8	0.0128 (2)	0.0150 (2)	0.0130 (2)	−0.00247 (18)	−0.00374 (18)	−0.00015 (18)
C9	0.0138 (2)	0.0146 (2)	0.0129 (2)	−0.00401 (18)	−0.00363 (17)	0.00107 (18)
C10	0.0129 (2)	0.0128 (2)	0.0116 (2)	−0.00302 (17)	−0.00291 (17)	0.00046 (17)
C11	0.0136 (2)	0.0159 (2)	0.0113 (2)	−0.00358 (18)	−0.00181 (17)	0.00037 (17)
C12	0.0124 (2)	0.0167 (2)	0.0125 (2)	−0.00295 (18)	−0.00149 (17)	−0.00021 (18)
C13	0.0127 (2)	0.0145 (2)	0.0126 (2)	−0.00236 (18)	−0.00379 (17)	0.00019 (18)
C14	0.0134 (2)	0.0191 (3)	0.0111 (2)	−0.00229 (19)	−0.00181 (17)	−0.00019 (18)
C15	0.0122 (2)	0.0177 (3)	0.0124 (2)	−0.00208 (18)	−0.00191 (17)	−0.00048 (18)
C16	0.0169 (2)	0.0151 (2)	0.0129 (2)	−0.00269 (19)	−0.00430 (18)	0.00006 (18)
C17	0.0206 (3)	0.0204 (3)	0.0173 (3)	−0.0031 (2)	−0.0091 (2)	0.0003 (2)

### Geometric parameters (Å, °)

**Table:**

O1—C7	1.2491 (7)	C9—C10	1.4543 (8)
O2—C1	1.3452 (8)	C9—H9A	0.93
O2—H1O2	0.928 (16)	C10—C15	1.3998 (8)
O3—C13	1.3621 (7)	C10—C11	1.4078 (8)
O3—C16	1.4334 (7)	C11—C12	1.3791 (8)
C1—C2	1.4006 (9)	C11—H11A	0.93
C1—C6	1.4174 (8)	C12—C13	1.4019 (8)
C2—C3	1.3803 (10)	C12—H12A	0.93
C2—H2A	0.93	C13—C14	1.3960 (8)
C3—C4	1.3993 (10)	C14—C15	1.3957 (8)
C3—H3A	0.93	C14—H14A	0.93
C4—C5	1.3868 (9)	C15—H15A	0.93
C4—H4A	0.93	C16—C17	1.5102 (9)
C5—C6	1.4053 (8)	C16—H16A	0.97
C5—H5A	0.93	C16—H16B	0.97
C6—C7	1.4808 (8)	C17—H17A	0.96
C7—C8	1.4632 (8)	C17—H17B	0.96
C8—C9	1.3475 (8)	C17—H17C	0.96
C8—H8A	0.93		
			
C1—O2—H1O2	105.5 (10)	C15—C10—C9	118.97 (5)
C13—O3—C16	118.56 (5)	C11—C10—C9	123.04 (5)
O2—C1—C2	117.49 (5)	C12—C11—C10	120.91 (5)
O2—C1—C6	122.28 (5)	C12—C11—H11A	119.5
C2—C1—C6	120.23 (6)	C10—C11—H11A	119.5
C3—C2—C1	120.32 (6)	C11—C12—C13	120.33 (5)
C3—C2—H2A	119.8	C11—C12—H12A	119.8
C1—C2—H2A	119.8	C13—C12—H12A	119.8
C2—C3—C4	120.34 (6)	O3—C13—C14	125.05 (5)
C2—C3—H3A	119.8	O3—C13—C12	114.98 (5)
C4—C3—H3A	119.8	C14—C13—C12	119.97 (5)
C5—C4—C3	119.71 (6)	C15—C14—C13	119.03 (5)
C5—C4—H4A	120.1	C15—C14—H14A	120.5
C3—C4—H4A	120.1	C13—C14—H14A	120.5
C4—C5—C6	121.34 (6)	C14—C15—C10	121.76 (5)
C4—C5—H5A	119.3	C14—C15—H15A	119.1
C6—C5—H5A	119.3	C10—C15—H15A	119.1
C5—C6—C1	118.06 (5)	O3—C16—C17	106.17 (5)
C5—C6—C7	122.80 (5)	O3—C16—H16A	110.5
C1—C6—C7	119.14 (5)	C17—C16—H16A	110.5
O1—C7—C8	120.46 (5)	O3—C16—H16B	110.5
O1—C7—C6	118.86 (5)	C17—C16—H16B	110.5
C8—C7—C6	120.67 (5)	H16A—C16—H16B	108.7
C9—C8—C7	119.61 (5)	C16—C17—H17A	109.5
C9—C8—H8A	120.2	C16—C17—H17B	109.5
C7—C8—H8A	120.2	H17A—C17—H17B	109.5
C8—C9—C10	127.21 (5)	C16—C17—H17C	109.5
C8—C9—H9A	116.4	H17A—C17—H17C	109.5
C10—C9—H9A	116.4	H17B—C17—H17C	109.5
C15—C10—C11	117.98 (5)		
			
O2—C1—C2—C3	−179.69 (6)	C7—C8—C9—C10	−178.73 (5)
C6—C1—C2—C3	0.31 (10)	C8—C9—C10—C15	173.80 (6)
C1—C2—C3—C4	0.31 (11)	C8—C9—C10—C11	−7.18 (10)
C2—C3—C4—C5	−0.43 (12)	C15—C10—C11—C12	0.63 (9)
C3—C4—C5—C6	−0.06 (11)	C9—C10—C11—C12	−178.39 (5)
C4—C5—C6—C1	0.65 (10)	C10—C11—C12—C13	0.49 (9)
C4—C5—C6—C7	179.82 (6)	C16—O3—C13—C14	0.40 (9)
O2—C1—C6—C5	179.22 (6)	C16—O3—C13—C12	−179.35 (5)
C2—C1—C6—C5	−0.78 (9)	C11—C12—C13—O3	178.52 (5)
O2—C1—C6—C7	0.03 (9)	C11—C12—C13—C14	−1.24 (9)
C2—C1—C6—C7	−179.97 (5)	O3—C13—C14—C15	−178.90 (6)
C5—C6—C7—O1	−175.37 (6)	C12—C13—C14—C15	0.83 (9)
C1—C6—C7—O1	3.79 (9)	C13—C14—C15—C10	0.32 (10)
C5—C6—C7—C8	5.26 (9)	C11—C10—C15—C14	−1.04 (9)
C1—C6—C7—C8	−175.59 (5)	C9—C10—C15—C14	178.03 (6)
O1—C7—C8—C9	3.15 (9)	C13—O3—C16—C17	179.71 (5)
C6—C7—C8—C9	−177.48 (5)		

### Hydrogen-bond geometry (Å, °)

**Table:**

*Cg*1 is the centroid of the O2,C1–C6 ring?

**Table:**

*D*—H···*A*	*D*—H	H···*A*	*D*···*A*	*D*—H···*A*
O2—H1O2···O1	0.93 (2)	1.66 (2)	2.5113 (7)	151 (1)
C16—H16A···Cg1^i^	0.97	2.70	3.5762 (7)	151
C16—H16B···Cg1^ii^	0.97	2.66	3.5339 (7)	151

Symmetry codes:  (i) −*x*+2, −*y*+1, −*z*; (ii) −*x*+2, −*y*+2, −*z*.
